# Glucagon-like peptide-1 receptor agonist exendin 4 ameliorates diabetes-associated vascular calcification by regulating mitophagy through the AMPK signaling pathway

**DOI:** 10.1186/s10020-024-00817-8

**Published:** 2024-05-08

**Authors:** Kui Chen, Hao-jie Jin, Zi-heng Wu, Bao-fu Zhang, Jun Wu, Zi-yi Huang, Ying-peng Huang, Xin-wu Lu, Xiang-tao Zheng

**Affiliations:** 1 Department of Vascular Surgery, The Second Affiliated Hospital of Wenzhou Medical University, 325015 Wenzhou, China; 2 Department of Vascular Surgery, The First Affiliated Hospital, School of Medicine, Zhejiang University, 310003 Hangzhou, China; 3 Department of Vascular Surgery, Shanghai Ninth People’s Hospital, Shanghai Jiao Tong University School of Medicine, 200011 Shanghai, China

**Keywords:** Glucagon-like peptide-1 receptor, Vascular calcification, Mitophagy, Mitophagosome-lysosome fusion, The AMPK signaling pathway

## Abstract

**Background:**

Vascular calcification (VC) is a complication in diabetes mellitus (DM) patients. Osteogenic phenotype switching of vascular smooth muscle cells (VSMCs) plays a critical role in diabetes-related VC. Mitophagy can inhibit phenotype switching in VSMCs. This study aimed to investigate the role of the glucagon-like peptide-1 receptor (GLP-1R) agonist exendin 4 (EX4) in mitophagy-induced phenotype switching.

**Materials and methods:**

The status of VC in T2DM mice was monitored using Von Kossa and Alizarin Red S (ARS) staining in mouse aortic tissue. Human aortic smooth muscle cells were cultured in high glucose (HG) and β-glycerophosphate (β-GP) conditioned medium. Accumulation of LC3B and p62 was detected in the mitochondrial fraction. The effect of EX4 in vitro and in vivo was investigated by knocking down AMPKα1.

**Results:**

In diabetic VC mice, EX4 decreased the percentage of von Kossa/ARS positive area. EX4 inhibited osteogenic differentiation of HG/β-GP-induced VSMCs. In HG/β-GP-induced VSMCs, the number of mitophagosomes was increased, whereas the addition of EX4 restored mitochondrial function, increased the number of mitophagosome-lysosome fusions, and reduced p62 in mitochondrial frictions. EX4 increased the phosphorylation of AMPKα (Thr172) and ULK1 (Ser555) in HG/β-GP-induced VSMCs. After knockdown of AMPKα1, ULK1 could not be activated by EX4. The accumulation of LC3B and p62 could not be reduced after AMPKα1 knockdown. Knockdown of AMPKα1 negated the therapeutic effects of EX4 on VC of diabetic mice.

**Conclusion:**

EX4 could promote mitophagy by activating the AMPK signaling pathway, attenuate insufficient mitophagy, and thus inhibit the osteogenic phenotype switching of VSMCs.

## Introduction

Vascular calcification (VC) is a common complication in patients with diabetes mellitus (DM), including T1DM and T2DM, and is a major cause of cardiovascular disease and death. Numerous studies have shown that vascular intimal calcification (VIC) and vascular medial calcification (VMC) are prevalent in the vasculature of patients with DM, but the mechanisms mediating the progression of VIC or VMC have not been elucidated. Current research suggests that transdifferentiation of vascular smooth muscle cells (VSMCs) into osteoblast-like cells, stimulated by high glucose and other factors, is central to the development of VMC (Durham et al. 2018). VSMCs are the major component of the medial vasculature and can switch from a contractile (quiescent) phenotype to a proliferative (osteogenic) phenotype. Osteogenic VSMCs are able to migrate into the intima, further proliferate, and increase extracellular matrix formation, becoming a major source of calcified matrix vesicles (Zhang et al. 2018).

In healthy VSMCs, dysfunctional mitochondria are removed by autophagosomes, a process known as mitochondrial autophagy (mitophagy). Recent studies have shown that mitophagy can inhibit phenotypic switching in VSMCs. For example, VSMC phenotypic switching induced by VC inducer [β-glycerophosphate (β-GP)] could be inhibited by promoting mitochondrial autophagy (Ma et al. 2019); overexpression of BCL2 interacting protein 3 was able to inhibit the VC inducer (lactate)-induced VSMC phenotypic switching by promoting mitochondrial autophagy (Zhu et al. 2019). Therefore, mitophagy helps to reduce osteogenic VSMCs, and it could also suppress VC by removing away the damaged mitochondria via mitophagy (Phadwal et al. 2021).

Insufficient mitophagy refers to cells with a reduced number of lysosomes, resulting in the accumulation of early autophagosomes. It is characterized by a partially increased autophagic flux, but due to the lack of a sufficient number of lysosomes, the process does not reach its full degradation capacity. Insufficient mitophagy can lead to impaired clearance of damaged mitochondria and intracellular accumulation of mitochondrial fragments, as well as increased production of reactive oxygen species (ROS). When mitophagy is insufficient, damaged mitochondria will accumulate, resulting in a gradual increase in the proportion of SQSTM1/p62 protein in total mitochondrial proteins. Therefore, the concomitant accumulation of p62 in the mitochondrial fraction is now widely recognized as a reflection of insufficient mitophagy (Ito et al. 2015). High glucose (HG) has been shown to result in insufficient mitophagy (Chen et al. 2018). Increased LC3B and p62 and decreased lysosomal content were also found in the vasculature of high-fat diet (HFD)-induced T2DM rats, suggesting that autophagic flux was blocked (Sun et al. 2021). Therefore, we hypothesized that insufficient mitophagy may be the main manifestation of impaired autophagy in diabetes-related VC.

Glucagon-like peptide-1 (GLP-1) is a hormone produced primarily by intestinal L-cells and belongs to the class of incretin hormones. The action of GLP-1 depends on its receptor, the GLP-1R. GLP-1R is widely expressed in various tissues of the body. Downregulation of GLP-1 has been found to be associated with calcific aortic valve disease (Xiao et al. 2021). GLP-1 or GLP-1R agonists improve osteogenic differentiation of smooth muscle cells in addition to their glucose-lowering effects (Lee and Jun 2014; Zhan et al. 2014, 2015). In addition, the GLP-1R agonist has been shown to improve diabetic atherosclerosis by regulating calcification of VSMCs (Shi et al. 2022). However, the mechanism of how GLP-1 regulates diabetes-associated VC remains unclear, and it remains to be investigated whether it directly affects VSMC osteogenic differentiation or indirectly by affecting blood glucose concentrations. Interestingly, GLP-1 and its related compounds can promote mitophagy (Lin et al. 2021; Yu et al. 2019) or inhibit mitophagy (Zhou et al. 2020) under different conditions, but GLP-1 analog or GLP-1R agonist both help to improve mitochondrial function (Moon et al. 2022; Kamiya et al. 2022; Lynch et al. 1987). Therefore, we hypothesized that GLP-1R agonist has a regulatory function in diabetes-associated VC for insufficient mitophagy in VSMCs.

In the current study, T2DM and VC mouse models were established, and the GLP-1R agonist exendin 4 (EX4) was used as a potential therapeutic agent for diabetes-related VC. Our data showed that EX4 attenuated VC in diabetic mice and inhibited osteogenic differentiation of vascular smooth muscle cells in HG medium. Meanwhile, we found that EX4 restored insufficient mitophagy by promoting mitophagosome formation and mitophagy, the molecular mechanism of which involved the adenosine 5’-monophosphate-activated protein kinase (AMPK) signaling pathway.

## Materials and methods

### Establishment of VC with diabetic mouse

C57BL/6 mice (6–7 weeks old) were bought from Changzhou Cavens Lab Animal Corporation (Changzhou, Jiangsu, China). As reported, HFD prior to streptozocin (STZ) administration can induce insulin resistance (Sun et al. 2021). HFD combined with STZ is commonly used to establish T2DM mouse models (Guo et al. 2022). After 3 weeks of HFD (60% fat, TP23300, Trophic Animal Feed High-Tech Co., Ltd, Nantong, Jiangsu, China), mice were intraperitoneally injected with STZ (HY-13,753, MedChemExpress Corporation, Shanghai, China) at 40 mg/kg/day for 5 consecutive days, and other mice were injected with citrate as the control for STZ. Blood glucose levels (in the tail vein) were measured with a glucometer. The level of fasting blood glucose was detected, which was more than 16.7 mmol/L was determined as T2DM mice. T2DM mice were divided into three groups: the DM group (*n* = 5), the DVC group (*n* = 5), and the DVC + EX4 group (*n* = 5). At the fifth week, mice in the DVC and DVC + EX4 groups were injected subcutaneously with VD2 (5 µL/g) for 2 days (Guo et al. 2022; Platko et al. 2020), while mice in the DM group were injected subcutaneously with phosphate-buffered saline (PBS, 5 µL/g) as the control for VD2. Subsequently, mice in the DVC + EX4 group were injected intraperitoneally with EX4 (24 nmol/kg/day, HY-13,443, MedChemExpress Corporation, Shanghai, China) for 26 consecutive days (Zhang et al. 2015; Yamane et al. 2011), and other mice were injected with the same volume of PBS as the control for EX4. The control mice (*n* = 5) were fed with control fat diet (CFD, LAD3001G, Trophic Animal Feed High-Tech Co., Ltd, Nantong, Jiangsu, China), injected intraperitoneally with the same volume of citrate and PBS. All the mice were euthanized at the ninth week. Thoracic aortic tissues were harvested for detection.

### Alkaline phosphatase (ALP) activity assay of aortic tissues

To measure calcium content, aortic tissues from the aortic arch to the iliac bifurcation were dissected and dried at 55 °C, and calcium was extracted with 10% formic acid overnight at 4 °C. Colorimetric quantification of calcium was achieved by a reaction with o-cresolphthalein, and total protein was determined by the Bradford protein assay (Luo et al. 2009).

### Von Kossa and alizarin red S (ARS) staining of aortic tissues

Von Kossa and ARS staining of aortic tissues were performed using the calcium staining kit (Von Kossa method, G3282, Solarbio Life Sciences, Beijing, China) and alizarin red S solution (G1450, Solarbio Life Sciences, Beijing, China). Aortic tissues were fixed in 10% neutral formalin prior to dehydration and embedding. Sections were embedded in 95% ethanol, placed vertically, and air-dried thoroughly. Von Kossa staining was performed according to the manufacturer’s instructions. For the ARS staining, the sections were then placed in a vat containing ARS solution and stained for 5–10 min followed by a quick rinse in distilled water. The sections were dehydrated in a conventional transparent manner and embedded in resin.

### Immunofluorescence staining

Aortic tissue was fixed in 4% paraformaldehyde, and permeabilized with 0.2% Triton X-100. Sections were stained for GLP-1R (green) with ab218532 (Abcam, Cambridge, UK) at 1/500 dilution, followed by AlexaFluor®488 goat anti-rabbit secondary antibody (ab150077, Abcam, Cambridge, UK) at 1/1000 dilution. Sections were also stained for α-SMA (red) with 67735-1-Ig (Proteintech, Wuhan, Hubei, China) at 1:200, followed by AlexaFluor®647 goat anti-mouse secondary antibody (ab150115, Abcam, Cambridge, UK). Nucleic acids were stained with 4’,6- diamidino‐2‐phenylindole (DAPI). Immunohistochemical signal intensity and positively stained field of tissue sections were evaluated using ImageJ software. Tomm20 antibody (ab283317, Abcam) and LAMP1 antibody (ab62562, Abcam) were used for cell immunofluorescence staining.

### Human aortic smooth muscle cell (HASMC) culture and induction of osteogenic differentiation

HASMCs were purchased from Procell Technology (CP-H081, Wuhan, Hubei, China). They were cultured in the complete medium of HASMCs (CM-H081, Procell Technology, Wuhan, Hubei, China) in an incubator at 37℃ and under 95% air and 5% CO_2_ conditions. To induce osteogenic differentiation, HASMCs were treated with 10 mM β-GP (Ma et al. 2020) in the absence or presence of high glucose (30 mM) (Ghasempour et al. 2022). In the β-GP + EX4 group, EX4 (100 nM) was added to the cell culture medium for 14 days (Takaku et al. 2021), and the calcium deposition was visualized using a calcium assay kit (ab102505, Abcam, Cambridge, UK) and ARS staining.

### Knockdown of GLP-1R in HASMCs

Small interfering RNA against GLP-1R (Si-Glp 1r) and the control siRNA were synthesized by RiboBio Technology, Guangzhou, Guangdong, China. Cell transfection was performed using Lipofextamin®RNAiMAX (Thermo Fisher Scientific, Waltham, MA, USA). Briefly, cells were seeded to reach 80% confluence at the time of transfection. Lipofextamin®RNAiMAX reagent was diluted in Opti-MEM® medium (Thermo Fisher Scientific, Waltham, MA, USA). Subsequently, siRNAs were also diluted in Opti-MEM® medium. The diluted siRNAs were added to the diluted Lipofextamin®RNAiMAX reagent (1:1 ratio). They were incubated at room temperature for 5 min. The siRNA-lipid complex was then added to HASMCs. After 48 h, transfected HASMCs were harvested for the induction of osteogenic differentiation and β-GP/EX4 treatments. Knockdown of AMPKα1 (Si-AMPKα1) in HASMCs was performed as described above.

### Western blotting

Cells were lysed on ice for 30 min in RIPA buffer (Thermo Fisher Scientific, Waltham, MA, USA) supplemented with 1 mM protease inhibitor phenylmethylsulfonyl fluoride (PMSF, Gibco, Waltham, MA, USA). Samples were boiled in protein loading buffer for 10 min at 100 °C in a metal bath, and equal amounts of proteins were separated by 10% sodium dodecyl sulfate-polyacrylamide gel electrophoresis (SDS-PAGE) gel and transferred to polyvinylidene fluoride (PVDF) membranes (Millipore, Bedford, USA). The membranes were then blocked in TRIS-buffered saline with 0.5% Tween 20 (TBST) containing 3% bovine serum albumin (BSA) for 1 h at room temperature, followed by incubation with primary antibodies at 4 °C overnight. Secondary antibodies (Goat Anti-Rabbit IgG H&L HRP, ab6721, 1:2000) were incubated for 1 h at room temperature, and the membrane signals were visualized using a chemiluminescent horseradish peroxidase (HRP) substrate reagent (Bio-Rad, Hercules, CA, USA), and images were captured using a Tanon5200 imaging system (Biotanon, Shanghai, China). β-Actin or Tomm20 was used as a control for cell lysate and mitochondrial fractions, respectively. The primary antibodies were shown as follows: anti-RUNX2 (1:200, 20700-1-AP Proteintech, Wuhan, Hubei, China), anti-BMP2 (1:1000, 15544-1-AP, Proteintech), anti-LC3B (1:1000, 14600-1-AP, Proteintech), anti-p62 (1:10000, ab109012, Abcam), anti-PINK1 (1:500, 23274-1-AP, Proteintech), anti-Parkin (1:1000, 14060-1-AP, Proteintech), anti-pAMPKα (T172) (1:1000, ab133448, Abcam), anti-AMPKα (1:1000, ab32047, Abcam), anti-pULK1 (S555) (1:1000, #5869, Cell Signaling Technology, Danvers, MA, USA), and anti-ULK1 (1 µg/mL, ab167139, Abcam).

### Mitochondrial function test

Mitochondrial membrane potential was detected with tetramethylrhodamine, methyl ester (TMRM, Thermo Fisher Scientific, Waltham, MA, USA) probe, and mitochondrial superoxide levels were detected with MitoSOX red (Thermo Fisher Scientific, Waltham, MA, USA). TMRM was diluted at 10 µM in media (10×) and aliquoted for single use. MitoSOX was also aliquoted for single use (Little et al. 2020). Relative fluorescence intensity (RFI) was calculated and analyzed using ImageJ software.

### Transmission electron microscopy (TEM)

Sections were fixed in 2.5% glutaraldehyde. Postfixation was performed in 1% (v/v) osmium tetroxide in PBS for 2 h at 4℃. Sections were then dehydrated through an acetone gradient and embedded in Araldite. Ultrathin sections were cut with a Leica Ultracut R ultramicrotome (Leica, Germany) and stained with lead citrate and uranyl acetate. Sections were observed under a JEM-1230 transmission electron microscope (JEOL, Japan).

### Mitochondrial fraction protein extraction

Mitochondrial fractions in mouse aortic tissue were isolated using the Mitochondria Isolation Kit for Tissue (89,801, Thermo Fisher Scientific, Waltham, MA, USA) according to the manufacturer’s instructions. Mitochondrial fractions in HASMCs were isolated using the Mitochondria Isolation Kit for Cultured Cells (89,874, Thermo Fisher Scientific, Waltham, MA, USA) according to the manufacturer’s instructions.

### Knockdown of AMPKα1 in vivo

Adeno-associated virus (AAV) 9 was used as the knockdown vector. The AMPKα1 knockdown vector (AAV-sh- AMPKα1) and the negative control (NC) vector (AAV-sh-NC) were established by Hanbio Technology (Shanghai, China). One day before VD2 injection, the AAV vectors (10^11^ vector genome copies/mouse) were administered by tail vein injection. Other treatments were the same as the described above. All the mice were euthanized at week 9. Thoracic aortic tissue was harvested for detection.

### Statistical analysis

All data are presented as mean ± standard deviation (SD). SPSS 26.0 (IBM, Armonk, NY, USA) and GraphPad Prism 9 were used for data analysis. ImageJ software was used for image analysis. The difference between two groups was compared by using the Student’s t-test. The difference between multiple groups was compared by using the one-way analysis of variance (ANOVA) followed by the Tukey’s post hoc test. Differences were considered statistically significant when *P* < 0.05.

## Results

### GLP-1R agonist EX4 attenuates VC in diabetic mice

**Fig. 1:**
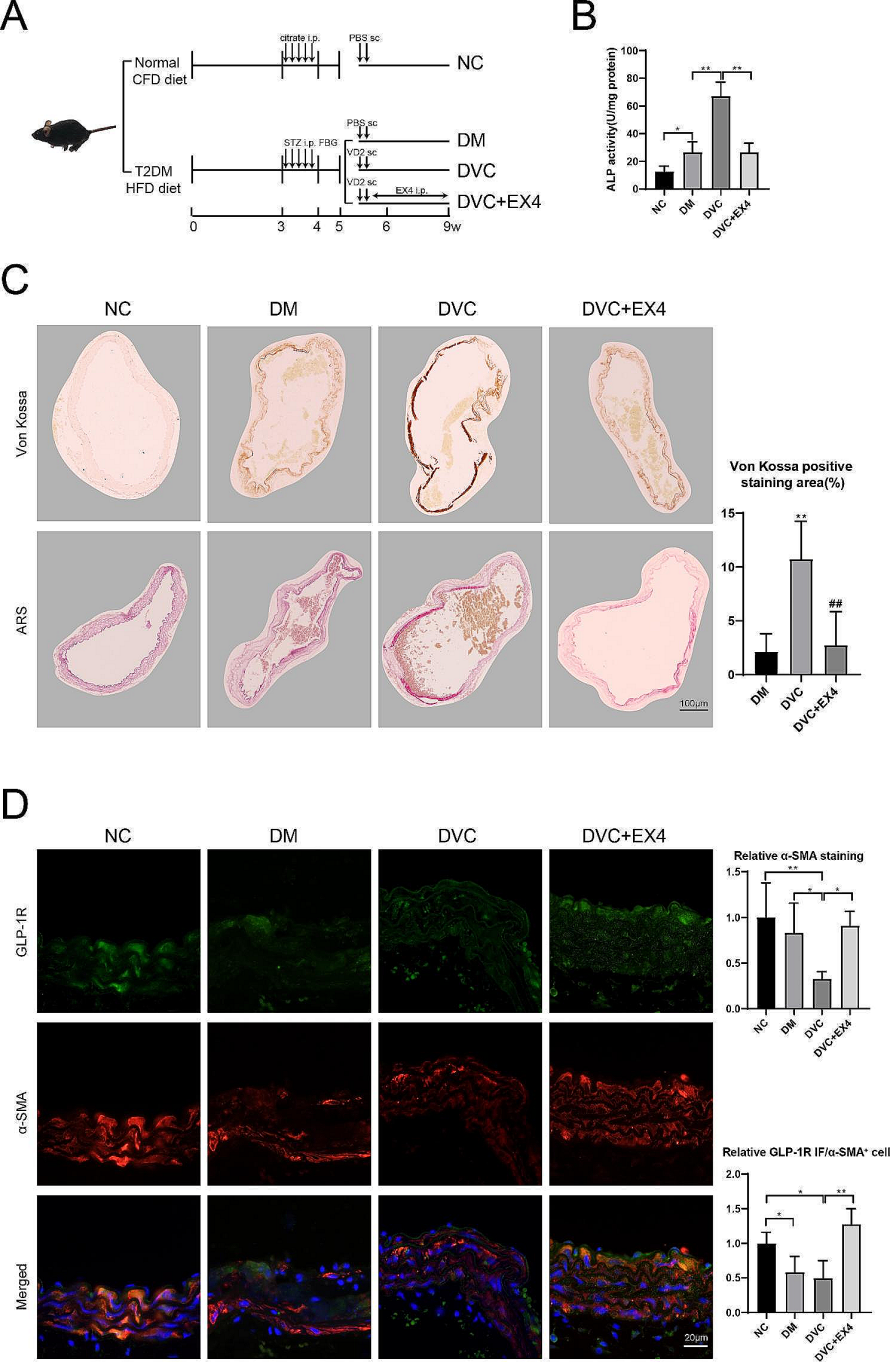
GLP-1R agonist EX4 attenuates VC in diabetic mice. (**A**) T2DM mouse model was induced by HFD and intraperitoneal injection of STZ in C57BL/6 mice. T2DM mice were divided into three groups: the DM group (*n* = 5), the DVC group (*n* = 5), and the DVC + EX4 group (*n* = 5). Mice in the DM group were injected subcutaneously with PBS, while mice in the DVC and DVC + EX4 groups were injected subcutaneously with VD2. Mice in the DVC + EX4 group were injected intraperitoneally with EX4 (24 nmol/kg/day) for 26 consecutive days. Control mice (*n* = 5) were fed with CFD, injected intraperitoneally with citrate, and injected subcutaneously with PBS. (**B**) ALP activity in aortic tissue was detected by a reaction with o-cresolphthalein. (**C**) Von Kossa and ARS staining of aortic tissue. **P < 0.01 vs. DM; ##*P* < 0.01 vs. DVC. (**D**) Immunofluorescence staining of aortic tissue with GLP-1R and α-SMA antibodies. *P < 0.05, **P < 0.01. Statistical analysis: mean ± SD, *N* = 5, one-way ANOVA with Tukey’s post hoc test

The T2DM mouse model was successfully established, and EX4 was administered as a therapeutic agent against VC in these diabetic mice (Fig. 1A). As a signature enzyme of mature osteoblasts, the ALP activity was elevated in the aortic tissue of DM and DVC mice, whereas the EX4 treatment reduced the ALP activity compared with the DVC group (Fig. 1B). Von Kossa and ARS staining of aortic tissue showed an increased positive area in DVC mice, whereas the EX4 treatment decreased the percentage of positive staining area (Fig. 1C). These data suggest that EX4 has the potential to attenuate VC in diabetic mice. In general, EX4 functions as a GLP-1R agonist by promoting downstream GLP-1R signaling pathways (Park et al. 2007). To determine whether EX4 directly mediated the signaling pathway of GLP-1R in VSMCs in aortic tissue, we characterized VSMCs with α-SMA and double-stained with GLP-1R (Fig. 1D), which showed that EX4 promoted the fluorescent signal of GLP-1R in VSMCs.

### EX4 inhibits osteogenic differentiation of HASMCs in HG medium

**Fig. 2:**
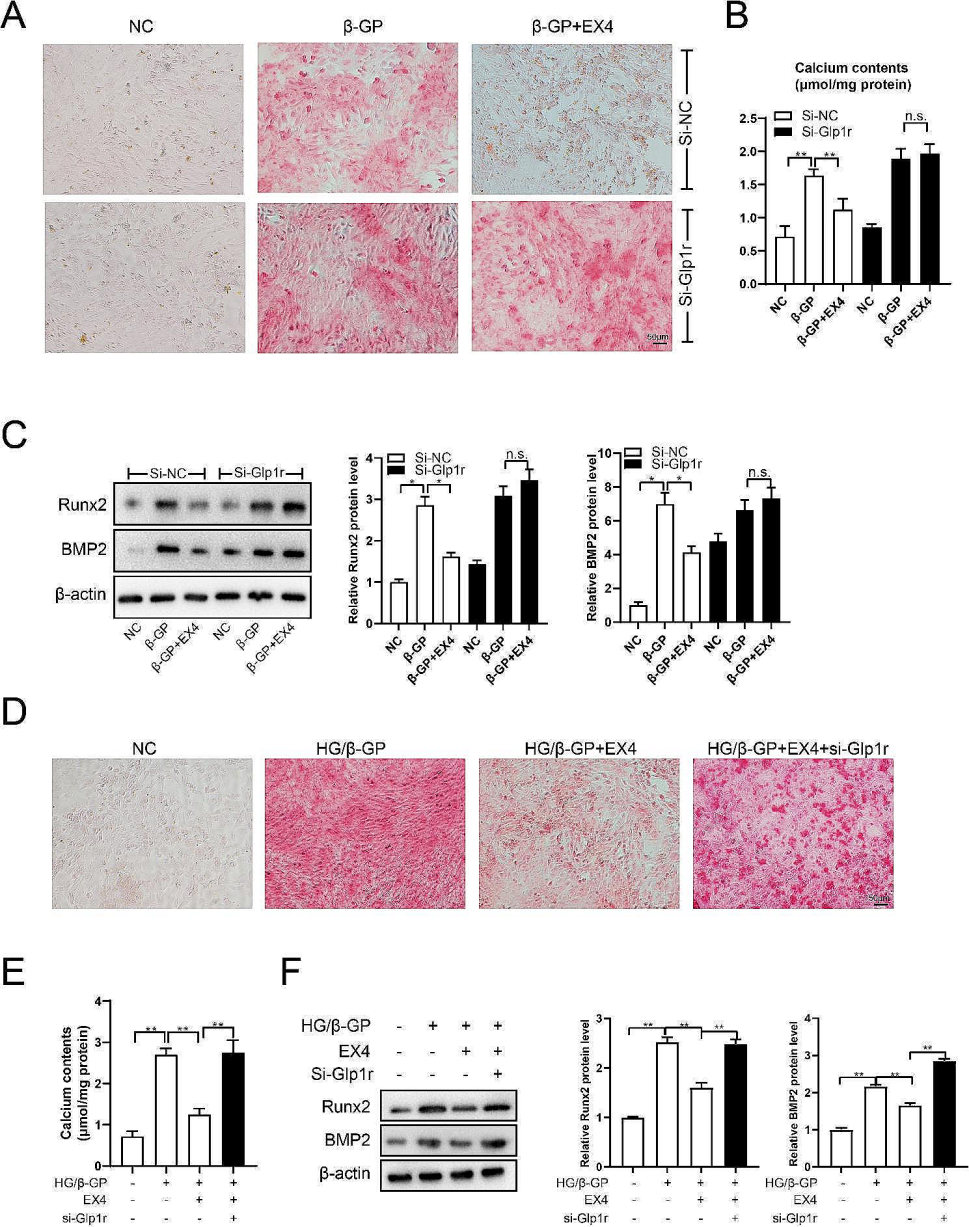
EX4 inhibits osteogenic differentiation of HASMCs in HG medium. To induce osteogenic differentiation, HASMCs were treated with 10 mM β-GP in the absence or presence of high glucose (30 mM). In the β-GP + EX4 group, EX4 (100 nM) was added to the cell culture medium for 14 days. GLP-1R knockdown was performed using siRNAs. After 48 h, transfected HASMCs were harvested for the induction of osteogenic differentiation and β-GP/EX4 treatments. (**A**) Calcium deposition was visualized by ARS staining. (**B**) Calcium content was detected using a calcium assay kit. (**C**) Osteogenic differentiation markers, including Runx2 and BMP2, were detected by Western blotting. (**D**) Calcium deposition was visualized by ARS staining in HG medium. (**E**) Calcium content was detected by calcium assay kit in HG medium. (**F**) Osteogenic differentiation markers, including Runx2 and BMP2, were detected by Western blotting in HG medium. **P < 0.01. Statistical analysis: mean ± SD, *N* = 3, one-way ANOVA with Tukey’s post hoc test

As reported, β-GP could induce the osteogenic differentiation of VSMCs (Alesutan et al. 2020; Niu et al. 2021). ARS staining showed that the addition of EX4 reduced the β-GP-induced osteogenic differentiation in HASMCs, whereas no such reduction was observed when GLP-1R expression was knocked down (Fig. 2A). Similarly, EX4 significantly reduced calcium content in β-GP-treated HASMCs, whereas no such reduction was observed when GLP-1R expression was knocked down (Fig. 2B). These data suggest that GLP-1R may mediate the effect of EX4 on osteogenic differentiation of VSMCs. Further detection of markers of VSMC osteogenic differentiation, including Runx2 and BMP2 (Guo et al. 2022), showed a similar trend with ARS staining and calcium content (Fig. 2C). We then used HG medium to mimic diabetic conditions in vitro. After GLP-1R knockdown in HASMCs, the effects of EX4 on inhibition of osteogenic differentiation (Fig. 2D), reduction of calcium content (Fig. 2E), and decrease of osteogenic markers (Fig. 2F) were negated.

### EX4 inhibits osteogenic differentiation of HASMCs via promoting mitophagy

**Fig. 3:**
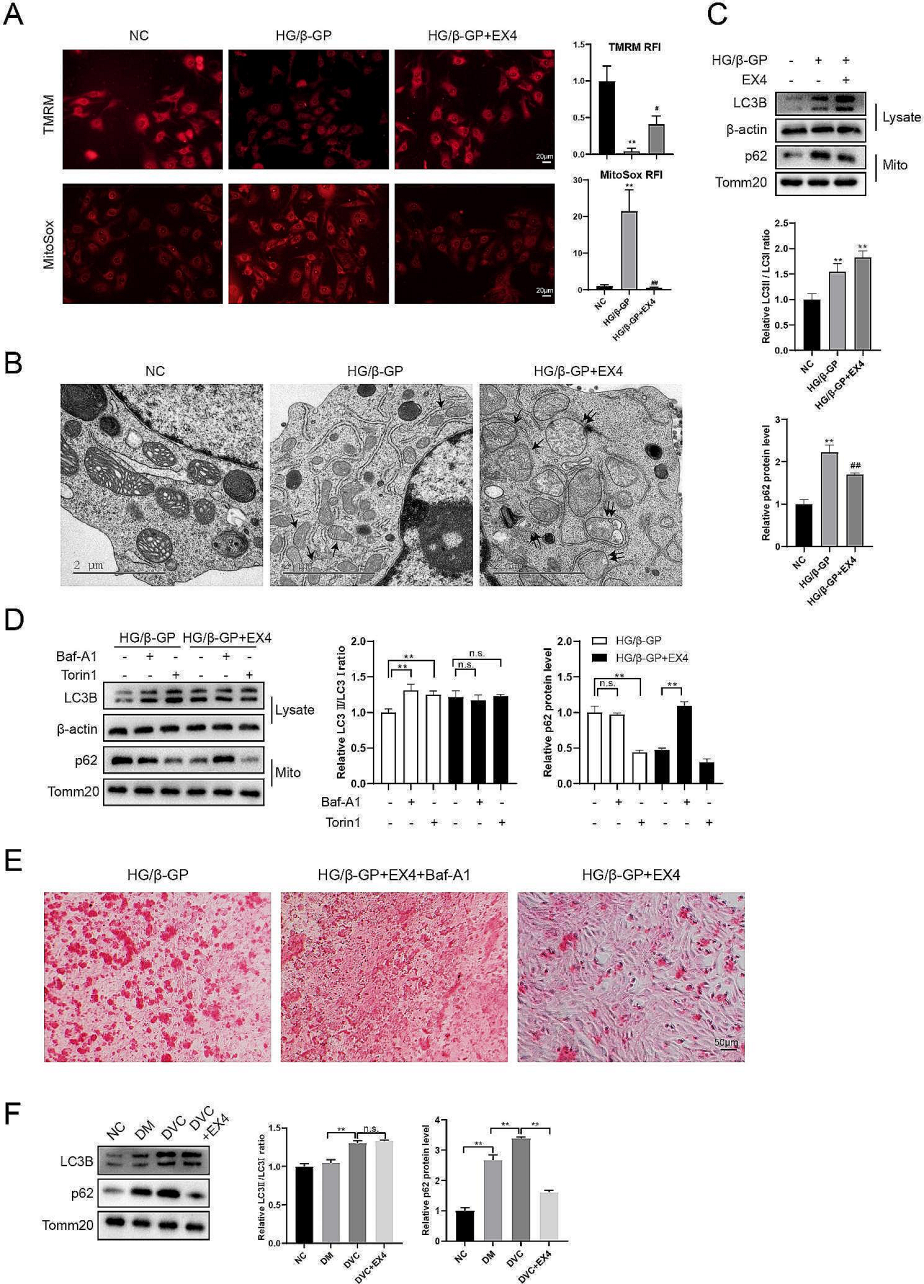
EX4 inhibits osteogenic differentiation of HASMCs via promoting mitophagy. To induce osteogenic differentiation, HASMCs were treated with 10 mM β-GP in HG medium (30 mM). In the β-GP + EX4 group, EX4 (100 nM) was added to the cell culture medium for 14 days. (**A**) Mitochondrial function tests were performed using TMRM probes and MitoSox red staining. **P < 0.01 vs. NC; #*P* < 0.05, ##*P* < 0.01 vs. HG/β-GP. (**B**) TEM was used to directly observe mitophagy. Mitophagosomes were indicated by single black arrows and mitophagosome-lysosome fusion was indicated by double black arrows. (**C**) LC3B expression in cell lysates and p62 expression in mitochondrial fractions were detected by Western blotting. (**D**) Autophagy/mitophagy inducer Torin1 (250 nM) or lysosome inhibitor Baf-A1 (20 nM) was added to the cell culture medium. LC3B expression in cell lysates and p62 expression in mitochondrial fractions were detected by Western blotting. (**E**) Calcium deposition was visualized by ARS staining. (**F**) Mitochondrial proteins were extracted from mouse aortic tissues, and the expressions of LC3B and p62 were detected by Western blotting. Statistical analysis: mean ± SD, *N* = 3, one-way ANOVA with Tukey’s post hoc test

To further investigate the molecular mechanism of EX4 on osteogenic differentiation, mitochondrial function tests were performed in HG/β-GP-treated HASMCs with or without EX4 treatment. In HG/β-GP-treated HASMCs, mitochondrial membrane potential was reduced, whereas they were reduced to normal by EX4 treatment (Fig. 3A). Meanwhile, mitochondrial superoxide levels were significantly upregulated in HG/β-GP-treated HASMCs, whereas it was reduced to normal by EX4 treatment (Fig. 3A). TEM was used to directly observe mitophagy. As shown in Fig. 3B, mitophagosomes (single black arrows) were increased in the HG/β-GP and HG/β-GP + EX4 groups compared with the NC group. In addition, mitophagosome-lysosome fusion (double black arrows) was higher in the HG/β-GP + EX4 group than in the HG/β-GP group. In cell lysates from the HG/β-GP and HG/β-GP + EX4 groups, LC3B levels were increased; however, in mitochondrial fractions, p62 levels in the HG/β-GP + EX4 group were lower than those in the HG/β-GP group (Fig. 3C). As the concomitant accumulation of p62 in mitochondrial fractions is now widely recognized as a reflection of insufficient mitophagy (Chen et al. 2018), it is suggested that EX4 may promote the insufficient mitophagy in HG/β-GP-treated HASMCs.

To further confirm this speculation, we added the autophagy/mitophagy inducer Torin1 (250 nM) or the lysosome inhibitor Baf-A1 (20 nM). The addition of Torin1 inhibited the accumulation of p62 in mitochondrial fractions, whereas the addition of Baf-A1 inhibited the ability of EX4 to downregulate p62 (Fig. 3D), again suggesting that EX4 could promote lysosome formation and accelerate p62 degradation. Meanwhile, the addition of Baf-A1 negated the effects of EX4 on osteogenic differentiation (Fig. 3E). In mitochondrial proteins extracted from mouse aortic tissues, p62 was accumulated in the DVC group, whereas it was reduced in the DVC + EX4 group (Fig. 3F). Taken together, these data suggested that EX4 inhibited osteogenic differentiation of HASMCs via promoting mitophagosome formation and mitophagy.

### EX4 promotes mitophagy via the AMPK signaling pathway

**Fig. 4:**
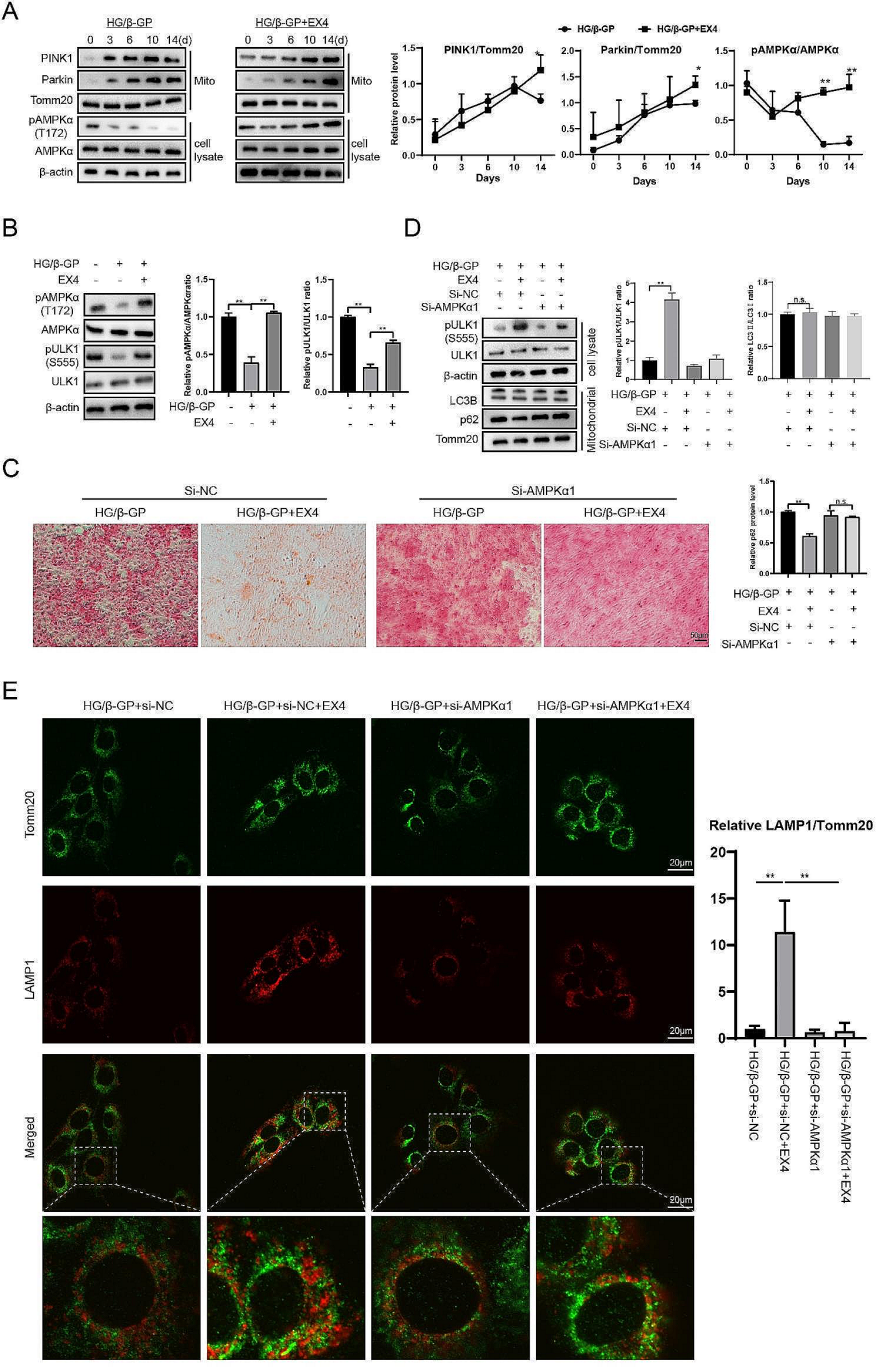
EX4 promotes mitophagy via the AMPK signaling pathway. To induce osteogenic differentiation, HASMCs were treated with 10 mM β-GP in HG medium (30 mM). In the β-GP + EX4 group, EX4 (100 nM) was added to the cell culture medium for 14 days. (**A**) The expressions of PINK1 and Parkin in mitochondrial fractions and the expressions of pAMPKα and AMPKα in cell lysates were detected by Western blotting. *P < 0.05, **P < 0.01 vs. HG/β-GP. (**B**) The expressions of pAMPKα, AMPKα, pULK1, and ULK1 were detected by Western blotting. (**C**) Knockdown of AMPKα1 was performed using siRNAs. Calcium deposition was visualized by ARS staining. (**D**) Expression of pULK1 and ULK1 in cell lysates and the expression of LC3B and p62 in mitochondrial fractions were detected by Western blotting. (**E**) Immunofluorescence staining of HASMCs using Tomm20 and LAMP1 antibodies. **P < 0.01. Statistical analysis: mean ± SD, *N* = 3, Student’s t-test (**A**) and one-way ANOVA with Tukey’s post hoc test (**B-E**)

PINK1 and Parkin play critical roles in mitophagosome formation (Uoselis et al. 2023). In consecutive 14D assays, we found that the changes of these two molecules in the mitochondrial fractions in the HG/β-Gp and HG/β-GP + EX4 groups were relatively similar without significant differences (Fig. 4A). Taken together with the results of Fig. 3, we suggest that the regulation of mitophagy by EX4 is mainly involved in promoting lysosomal binding to autophagosomes, and we therefore consider other pathways. Knockdown of AMPK is related to the accumulation of mitophagic p62 (Qiang et al. 2013). In addition, GLP-R1 agonist could activate the AMPK signaling (Kamiya et al. 2022). As shown in Fig. 4A, EX4 maintained the activation of AMPK (Thr172 phosphorylation). The activation of ULK1 (Ser555 phosphorylation), a downstream molecule of AMPK, was well maintained by EX4 (Fig. 4B). When AMPK was knocked down, EX4 was deprived of its inhibitory function for osteogenic differentiation of HASMCs (Fig. 4C). At this time point, the phosphorylation level of ULK1 was attenuated and the accumulation of p62 in mitochondrial fractions was still present (Fig. 4D). After co-staining with Tomm20 and LAMP1 (lysosomal marker), the AMPK knockdown group was unable to maintain the lysosomal-promoting effect of EX4, as evidenced by a decrease in merged dots (Fig. 4E). As reported, ULK1 is required for mitophagy at the step of lysosomal targeting of damaged/dysfunctional mitochondria (Laker et al. 2017). Our data indicated that EX4 alleviated the impaired mitophagy induced by HG/β-GP via the AMPK signaling pathway.

**Fig. 5:**
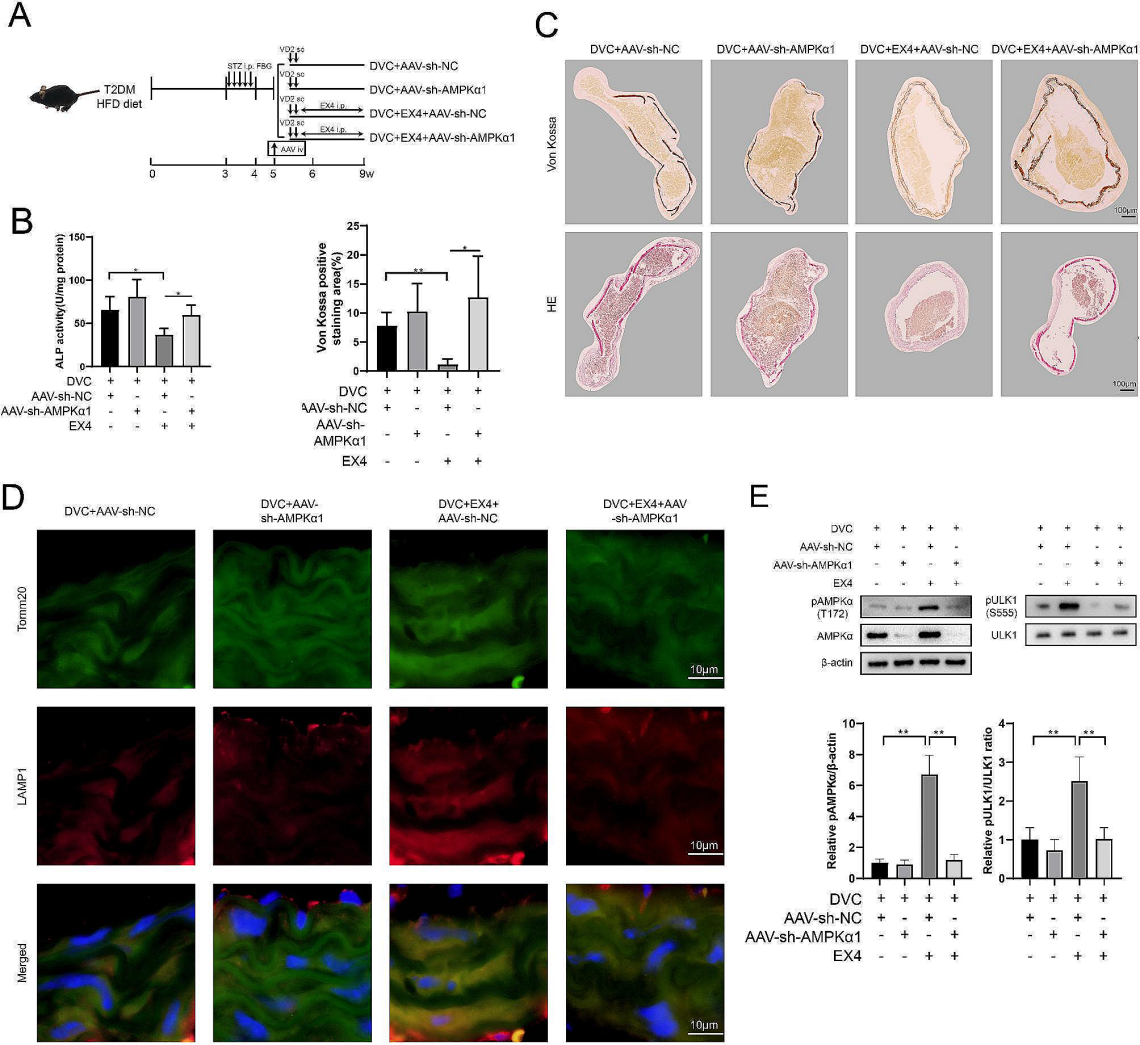
Inhibition of VC by EX4 is attenuated byin vivo inhibition of AMPK. (**A**) AAV9 was used as a knockdown vector for AMPKα1 in vivo. One day before VD2 injection, the AAV vectors (10^11^ vector genome copies/mouse) were administered by tail vein injection. Other treatments were the same as Fig. 1. (**B**) ALP activity in aortic tissue was detected by reaction with o-cresolphthalein. (**C**) Von Kossa and ARS staining of aortic tissue. (**D**) Immunofluorescence staining of aortic tissue with Tomm20 and LAMP1 antibodies. (**E**) Expression of pULK1, ULK1, pAMPKα, and AMPKα were detected by Western blotting. *P < 0.05, **P < 0.01. Statistical analysis: mean ± SD, *N* = 6, one-way ANOVA with Tukey’s post hoc test (**B, E**) and Welch’s ANOVA with Games-Howell’s post hoc test (**C**)

In the in vivo experiments, AAV9 vectors were used to knock down AMPK in diabetic mice (Fig. 5A). The results of aortic ALP activity assay (Fig. 5B), Von Kossa staining, and ARS staining (Fig. 5C) showed that the therapeutic effects of VC by EX4 were attenuated after inhibition of AMPK in vivo. Localization of lysosomes to mitochondria in aortic tissue by co-staining with anti-LAMP1 and anti-TOMM20 showed that EX4 increased the content of lysosomes around mitochondria, whereas the change was not significant after AMPK knockdown (Fig. 5D). These results indicated that, EX4 promoted mitophagy processing through the AMPK pathway, which was consistent with the in vitro experiments. Meanwhile, Western blotting assays performed on the tissues also demonstrated the regulation of EX4 for the AMPK signaling pathway (Fig. 5E).

## Discussion

**Fig. 6:**
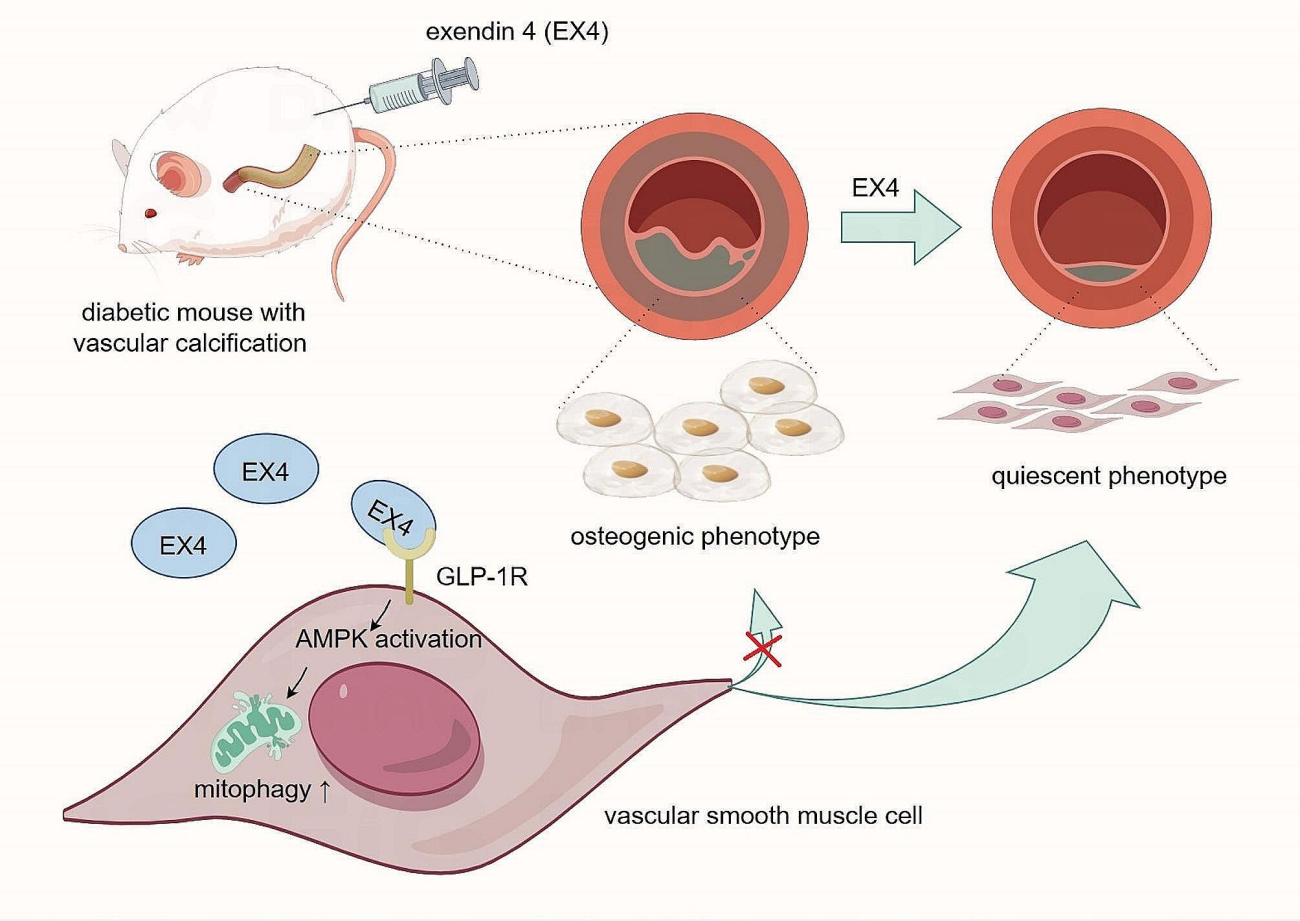
As a promoter of mitophagy, EX4 inhibits osteogenic differentiation of VSMCs by promoting mitophagy through the AMPK signaling pathway, thus attenuating DM-related VC.

Osteogenic phenotype switching of VSMCs plays a critical role in diabetic-related VC. During such phenotype switching, the molecular regulatory mechanisms underlying insufficient mitophagy remain unclear. The current study focused on the mechanisms of insufficient mitophagy in HG/β-GP-induced VSMCs, and investigated the role of GLP-1R agonist—EX4—in regulating mitophagy. Our data showed that EX4, as a promoter of mitophagy, could inhibit osteogenic differentiation of VSMCs in HG medium. In addition, EX4 promoted mitophagy through the AMPK signaling pathway (Fig. 6). This study verified for the first time the regulatory role of EX4 in HG/β-GP-induced VSMCs, suggesting that EX4 has a potential in the treatment of VC in diabetic patients.

The maintenance of mitochondrial quality is achieved by a precise balance between biogenesis and degradation processes that facilitate cellular renewal. Mitochondria are highly dynamic organelles that continuously undergo cycles of fusion and fission (Passos and Zglinicki 2012). Fusion events have been shown to rescue impaired mitochondria by redistributing proteins and maintaining mitochondrial DNA integrity (Westermann 2010). In cases of more severe damage, fission-mediated segregation of irreversibly impaired mitochondria becomes a prerequisite for their appropriate engulfment and subsequent degradation via mitophagy, a selective autophagy process specific to mitochondria (Rubinsztein et al. 2011). Thus, the coordinated regulation of mitochondrial biogenesis, dynamics, and degradation by mitophagy represents a set of quality control mechanisms aimed at preventing the accumulation of damaged mitochondria and the excessive production of ROS (López-Otín et al. 2013). As a highly conserved mechanism, mitophagy selectively delivers unwanted mitochondria for lysosomal degradation (Rubinsztein et al. 2011). Stress-induced membrane depolarization stabilizes PINK1, then the E3 ubiquitin ligase PARK2 is recruited to the mitochondria. Binding of the receptor protein p62 requires the participation of PARK2-mediated ubiquitination of mitochondrial substrates. This process can recognize both ubiquitinated LC3 and substrates on phagophores, the precursors of autophagosomes (Green et al. 2011). Therefore, it is now widely accepted that the concomitant accumulation of p62 reflects, at least in part, insufficient autophagy. In the current study, we used mitochondrial function assay and TEM to observe the mitophagy status of VSMCs. In HG/β-GP-induced VSMCs, mitochondrial function was dysregulated as evidenced by decreased TMRM staining and increased MitoSox staining. TEM revealed an increased number of mitophagosomes. These data indicated that HG resulted in the increase of insufficient mitophagy, which is consistent with the study of Chen et al. (Chen et al. 2018). Meanwhile, the accumulation of p62 in the mitochondrial frictions of HG/β-GP-induced VSMCs suggested the block of autophagic flux, which was also shown in the study of Sun et al. (Sun et al. 2021). After the addition of EX4, mitochondrial function was restored, the number of mitophagosome-lysosome fusions was increased, and the expression of p62 in mitochondrial frictions was reduced. These data indicated that EX4 could promote mitochondrial autophagy by increasing mitophagosome-lysosome fusion.

At the onset of autophagy, activated AMPK promotes the phosphorylation of ULK1 by (1) directly phosphorylating ULK1; (2) reducing the phosphorylation inhibition of ULK1 by mTOR complex 1 (mTORC1), which in turn promotes the expression of downstream autophagy marker proteins (genes essential for autophagy, ATG), including ATG4, ATG7, ATG3 (LC3A), and LC3B. Thus, the activation of AMPK induces autophagy and autophagic flux. Knockdown of AMPK leads to accumulation of intracellular p62 (Qiang et al. 2013). In HG/β-GP-induced VSMCs, the phosphorylation of ULK1 (Ser555) was decreased, indicating the low level of mitophagy. It was promoted by EX4 treatment, indicating that EX4 may promote mitophagy through the activation of AMPK. A previous study showed that application of the GLP-1R agonist EX4 in skeletal muscle cells activated the AMPK pathway (Wu et al. 2022). Another GLP-1R agonist, PF1801, inhibited muscle fiber necroptosis by activating the AMPK signaling pathway (Kamiya et al. 2022). Consistent with their study, EX4 increased the phosphorylation of AMPKα (Thr172) and ULK1 (Ser555) in HG/β-GP-induced VSMCs. After AMPKα1 knockdown, ULK1 could not be activated by EX4. In addition, the accumulation of LC3B and p62 could not be reduced after AMPKα1 knockdown. These data indicated that EX4 promoted mitophagy through the AMPK signaling pathway. Next, in the in vivo experiments, we knocked down AMPKα1 using AAV vectors. The results showed that knockdown of AMPKα1 negated the therapeutic effects of EX4 on diabetic mouse VC.

In conclusion, this study focused on the therapeutic mechanisms of GLP-1R agonist EX4 on diabetic-related VC. Our results showed that EX4 could promote mitophagy by activating the AMPK signaling pathway, attenuate insufficient mitophagy, and thus inhibit osteogenic differentiation of VSMCs. Our study highlighted this novel insight into the potential effects of EX4 in the field of prevention of diabetes complications.

## Data Availability

The datasets used and/or analyzed during the current study are available from the corresponding author on reasonable request.

## Abbreviations

VC: Vascular calcification
DM: Diabetes mellitus
VIC: Vascular intimal calcification
VMC: Vascular medial calcification
VSMC: Vascular smooth muscle cell
β-GP: β-glycerophosphate
ROS: Reactive oxygen species
HG: High glucose
HFD: High-fat diet
GLP-1: Glucagon-like peptide-1
EX4: Exendin 4
AMPK: Adenosine 5’-monophosphate-activated protein kinase
STZ: Streptozocin
PBS: Phosphate-buffered saline
CFD: Control fat diet
ALP: Alkaline phosphatase
ARS: Alizarin Red S
DAPI: 4’,6- diamidino‐2‐phenylindole
HASMC: Human aortic smooth muscle cell
PMSF: Phenylmethylsulfonyl fluoride
SDS-PAGE: Sodium dodecyl sulfate-polyacrylamide gel electrophoresis
TBST: TRIS-buffered saline with 0.5% Tween 20
BSA: Bovine serum albumin
HRP: Horseradish peroxidase
TMRM: tetramethylrhodamine, methyl ester
RFI: Relative fluorescence intensity
TEM: Transmission electron microscopy
AAV: Adeno-associated virus
NC: Negative control
SD: Standard deviation
ANOVA: Analysis of variance

## References

[CR30] Alesutan I, Moritz F, Haider T (2020). Impact of β-glycerophosphate on the bioenergetic profile of vascular smooth muscle cells. J Mol Med (Berl).

[CR7] Chen K, Dai H, Yuan J (2018). Optineurin-mediated mitophagy protects renal tubular epithelial cells against accelerated senescence in diabetic nephropathy. Cell Death Dis.

[CR1] Durham AL, Speer MY, Scatena M (2018). Role of smooth muscle cells in vascular calcification: implications in atherosclerosis and arterial stiffness. Cardiovasc Res.

[CR26] Ghasempour G, Mohammadi A, Zamani-Garmsiri F (2022). Upregulation of TGF-β type II receptor in high glucose-induced vascular smooth muscle cells. Mol Biol Rep.

[CR39] Green DR, Galluzzi L, Kroemer G (2011). Mitochondria and the autophagy-inflammation-cell death axis in organismal aging. Science.

[CR20] Guo B, Shan SK, Xu F (2022). Protective role of small extracellular vesicles derived from HUVECs treated with AGEs in diabetic vascular calcification. J Nanobiotechnol.

[CR6] Ito S, Araya J, Kurita Y (2015). PARK2-mediated mitophagy is involved in regulation of HBEC senescence in COPD pathogenesis. Autophagy.

[CR18] Kamiya M, Mizoguchi F, Yasuda S (2022). Amelioration of inflammatory myopathies by glucagon-like peptide-1 receptor agonist via suppressing muscle fibre necroptosis. J Cachexia Sarcopenia Muscle.

[CR34] Laker RC, Drake JC, Wilson RJ (2017). Ampk phosphorylation of Ulk1 is required for targeting of mitochondria to lysosomes in exercise-induced mitophagy. Nat Commun.

[CR10] Lee YS, Jun HS (2014). Anti-diabetic actions of glucagon-like peptide-1 on pancreatic beta-cells. Metabolism.

[CR14] Lin TK, Lin KJ, Lin HY (2021). Glucagon-like Peptide-1 receptor agonist ameliorates 1-Methyl-4-Phenyl-1,2,3,6-Tetrahydropyridine (MPTP) neurotoxicity through enhancing Mitophagy Flux and reducing α-Synuclein and oxidative stress. Front Mol Neurosci.

[CR28] Little AC, Kovalenko I, Goo LE (2020). High-content fluorescence imaging with the metabolic flux assay reveals insights into mitochondrial properties and functions. Commun Biol.

[CR38] López-Otín C, Blasco MA, Partridge L (2013). The hallmarks of aging. Cell.

[CR24] Luo XH, Zhao LL, Yuan LQ (2009). Development of arterial calcification in adiponectin-deficient mice: adiponectin regulates arterial calcification. J Bone Min Res.

[CR19] Lynch SC, Eckert PJ, Gruenwedel DW (1987). Induction of DNA repair in HeLa S3 carcinoma cells by the N-nitroso derivatives of 1-(N-L-tryptophan)-1-deoxy-D-fructose and 1-(5-hydroxytryptamino)-1-deoxy-D-fructose. IARC Sci Publ.

[CR3] Ma WQ, Sun XJ, Wang Y (2019). Restoring mitochondrial biogenesis with metformin attenuates β-GP-induced phenotypic transformation of VSMCs into an osteogenic phenotype via inhibition of PDK4/oxidative stress-mediated apoptosis. Mol Cell Endocrinol.

[CR25] Ma WQ, Sun XJ, Zhu Y (2020). PDK4 promotes vascular calcification by interfering with autophagic activity and metabolic reprogramming. Cell Death Dis.

[CR17] Moon JS, Hong JH, Jung YJ (2022). SGLT-2 inhibitors and GLP-1 receptor agonists in metabolic dysfunction-associated fatty liver disease. Trends Endocrinol Metab.

[CR31] Niu J, Wu C, Zhang M (2021). κ-opioid receptor stimulation alleviates rat vascular smooth muscle cell calcification via PFKFB3-lactate signaling. Aging.

[CR29] Park CW, Kim HW, Ko SH (2007). Long-term treatment of glucagon-like peptide-1 analog exendin-4 ameliorates diabetic nephropathy through improving metabolic anomalies in db/db mice. J Am Soc Nephrol.

[CR35] Passos JF, Zglinicki T (2012). Mitochondrial dysfunction and cell senescence–skin deep into mammalian aging. Aging.

[CR5] Phadwal K, Vrahnas C, Ganley IG (2021). Mitochondrial dysfunction: cause or consequence of vascular calcification?. Front Cell Dev Biol.

[CR21] Platko K, Lebeau PF, Gyulay G (2020). TDAG51 (T-Cell death-Associated Gene 51) is a key modulator of vascular calcification and osteogenic transdifferentiation of arterial smooth muscle cells. Arterioscler Thromb Vasc Biol.

[CR33] Qiang L, Wu C, Ming M (2013). Autophagy controls p38 activation to promote cell survival under genotoxic stress. J Biol Chem.

[CR37] Rubinsztein DC, Mariño G, Kroemer G (2011). Autophagy and aging. Cell.

[CR13] Shi LL, Hao M, Jin ZY (2022). Liraglutide alleviates Diabetic atherosclerosis through regulating calcification of vascular smooth muscle cells. Dis Markers.

[CR8] Sun XJ, Ma WQ, Zhu Y (2021). POSTN promotes diabetic vascular calcification by interfering with autophagic flux. Cell Signal.

[CR27] Takaku S, Tsukamoto M, Niimi N et al. Exendin-4 promotes Schwann Cell Survival/Migration and Myelination in Vitro. Int J Mol Sci. 2021;22(6). 10.3390/ijms22062971 PMC7999558 33804063

[CR32] Uoselis L, Nguyen TN, Lazarou M (2023). Mitochondrial degradation: Mitophagy and beyond. Mol Cell.

[CR36] Westermann B (2010). Mitochondrial fusion and fission in cell life and death. Nat Rev Mol Cell Biol.

[CR40] Wu L, Zhou M, Li T (2022). GLP-1 regulates exercise endurance and skeletal muscle remodeling via GLP-1R/AMPK pathway. Biochim Biophys Acta Mol Cell Res.

[CR9] Xiao F, Zha Q, Zhang Q (2021). Decreased Glucagon-Like Peptide-1 is Associated with Calcific aortic valve disease: GLP-1 suppresses the calcification of aortic valve interstitial cells. Front Cardiovasc Med.

[CR23] Yamane S, Hamamoto Y, Harashima S (2011). GLP-1 receptor agonist attenuates endoplasmic reticulum stress-mediated β-cell damage in Akita mice. J Diabetes Investig.

[CR15] Yu X, Hao M, Liu Y (2019). Liraglutide ameliorates non-alcoholic steatohepatitis by inhibiting NLRP3 inflammasome and pyroptosis activation via mitophagy. Eur J Pharmacol.

[CR12] Zhan JK, Tan P, Wang YJ (2014). Exenatide can inhibit calcification of human VSMCs through the NF-kappaB/RANKL signaling pathway. Cardiovasc Diabetol.

[CR11] Zhan JK, Wang YJ, Wang Y (2015). The protective effect of GLP-1 analogue in arterial calcification through attenuating osteoblastic differentiation of human VSMCs. Int J Cardiol.

[CR22] Zhang E, Xu F, Liang H (2015). GLP-1 receptor agonist Exenatide attenuates the detrimental effects of obesity on Inflammatory Profile in Testis and sperm quality in mice. Am J Reprod Immunol.

[CR2] Zhang C, Zhang K, Huang F (2018). Exosomes, the message transporters in vascular calcification. J Cell Mol Med.

[CR16] Zhou HR, Ma XF, Lin WJ (2020). Neuroprotective role of GLP-1 Analog for Retinal Ganglion cells via PINK1/Parkin-Mediated Mitophagy in Diabetic Retinopathy. Front Pharmacol.

[CR4] Zhu Y, Ji JJ, Yang R (2019). Lactate accelerates calcification in VSMCs through suppression of BNIP3-mediated mitophagy. Cell Signal.

